# High-flow oxygen therapy in cluster headache patients has no significant effect on nociception specific blink reflex parameters: a pilot study

**DOI:** 10.1186/s10194-016-0597-x

**Published:** 2016-02-12

**Authors:** D. Y. P. Haane, A. Plaum, P. J. Koehler, M. P. W. A. Houben

**Affiliations:** Department of Neurology and Clinical Neurophysiology, Zuyderland Medical Center Heerlen, P.O. Box 5500, 6130 MB Sittard, The Netherlands; Current address: Department of Neurology, Mariaziekenhuis Noord-Limburg, Overpelt, Belgium; Current address: Faculty of Health, Medicine and Life Sciences, Maastricht University, Maastricht, The Netherlands

**Keywords:** Cluster headache, Nociception specific blink reflex, Oxygen therapy, Normal values

## Abstract

**Background:**

The exact pathophysiology of cluster headache is unclear. We examined the influence of interneurons on the trigemino-facial reflex arch and the effect of oxygen, by using the nociception specific blink reflex parameters.

**Findings:**

There is no significant effect of oxygen, immediately and over time, on the nociception specific blink reflex parameters in ten male patients during the active phase of cluster headache, outside attacks. Also, there is no significant difference between the symptomatic and asymptomatic side. None of the subjects experienced a cluster headache attack during study participation. We therefore present the collected data as reference values of nociception specific trigeminal stimulation and the effect of oxygen on nociception specific blink reflex parameters.

**Conclusion:**

The nociception specific blink reflex seems not a suitable instrument for exploring the pathophysiology of cluster headache.

## Introduction

The exact pathophysiology of cluster headache (CH) is unclear. Previous studies have shown that 100% oxygen (O_2_) therapy is a notable CH attack reliever [1]. Exactly how oxygen exerts its pain reducing effect in patients with CH is uncertain, but it is shown to directly or indirectly cause vasoconstriction. Indirect vasoconstriction can be the result of a possible action on the parasympathetic outflow from the superior salivatory nucleus, as is shown in rats [2].

The blink reflex (BR) is a brainstem reflex, elicited through stimulation of the supraorbital nerve, derived from the first branch of the trigeminal nerve, resulting in a bilateral blink reaction of the eyelids through the facial nerve. The BR is composed of an early pontine response (R1), and a late medullary response (R2) [3]. R1 is oligosynaptic, ipsilateral and not clinically visible, whereas R2 is polysynaptic, bilateral and clinically observable [4]. A nociception specific blink reflex (nBR) can be elicited by transcutaneously selectively stimulating superficial nociceptive A-delta fibers of the supraorbital nerve with a concentric planar stimulating electrode. The response consists of only a bilateral R2. Using the nBR, the function of the afferent trigeminal and efferent facial nerves and their central connections can be assessed [3].

We wanted to examine the influence of interneurons on the trigemino-facial reflex arch and the effect of high-flow (12 liter/minute (L/min)) O_2_ by using the nBR and its parameters. However, none of the subjects experienced a CH attack during study participation, despite the fact that all of the subjects were in a cluster period at the time. This was possibly due to a preventive effect of nociception specific trigeminal stimulation on CH attacks [5]. We therefore present the data as reference values of the nBR parameters in patients in a cluster period outside a CH attack and the effect of high-flow O_2_ inhalation.

### Methods

Information concerning study population, in- and exclusion, equipment and questionnaires was already described in a previous publication [5]. The study was approved by the local ethics committee. All patients gave written informed consent. The study terminated early because none of the patients experienced a CH attack during clinical study time. One patient was excluded because the diagnosis of CH was questioned following study participation. CH patients were not compared to healthy controls; the baseline measurement was considered a control.

We elicited nBRs in eleven patients using Synergy EMG equipment (Natus Neurology). For stimulation we used a concentric planar electrode with central cathode and external anode ring (K2 concentric ring stimulating electrode, 1.5 mm; Inomed, Emmendingen, Germany). Disposable silver/silver chloride electrodes were placed over the orbicularis oculi muscles, just lateral of the mid-pupillary line (active) and near the lateral canthus (reference). The ground electrode was placed on the chin. The supraorbital nerve was stimulated ten millimeter cranial of the supraorbital notch with a 200 pulse per second (pps) train of three 0.5 ms pulses. The current intensity was increased stepwise by 0.3 mA, with regard to the tolerance limit of the patient, up to 20 % above the level that acquired stable R2 responses to assure supramaximal stimulation, with a maximum of 2.1 mA (once 2.4 mA). The stimuli were delivered at unpredictable intervals of at least 15 s to minimize habituation. Both the symptomatic and the asymptomatic side were stimulated until we had obtained four blink reflexes on each side (here referred to as one measurement). In each subject, the R2 responses were elicited at at least the five time points: before O_2_ inhalation, during O_2_ inhalation and every two hours thereafter up until six hours after O_2_ inhalation. It was originally planned to continue until a spontaneous CH attack occurred, but this did not happen.

We analyzed the measurements before, during and six hours after O_2_ inhalation. All responses were evaluated by two researchers (DH and MH). For each stimulation site and time we calculated the shortest latency, amplitude, duration and area of the R2 response using Synergy Reader version 20.1.0.100 (Natus Neurology).

#### Statistical analysis

We performed the analyses using IBM SPSS statistics version 21. Variables were tested for normal distribution (Shapiro-Wilk). We calculated mean with standard deviation or median with interquartile range as appropriate. Differences of mean were tested with a paired samples t-test. Differences of median were tested using Wilcoxon signed-rank test. Significance levels were adjusted for multiple testing by Bonferroni correction (*p* < 0.0025).

### Results

Ten CH patients were included. All CH patients were men. Mean age was 45.7 (range 24–69). Mean BMI was 24.0 (range 20.5–36.0). Three patients had episodic CH, five patients had chronic CH and two patients were in their first cluster. Six patients experienced attacks on the left side, four on the right. Eight patients were current smokers.

Table 1 shows the nBR parameters of the symptomatic and asymptomatic side after both ipsilateral and contralateral stimulation, and before and during O_2_ inhalation (*n* = 10). There is no significant difference in the nBR parameters before and during O_2_ inhalation. There were also no differences in baseline parameters when the symptomatic side was compared to the asymptomatic side. We then studied the difference between the measurements before O_2_ inhalation and six hours after O_2_ inhalation (*n* = 9; the measurement in one subject was rejected because it was impossible to elicit R2 responses after six hours). This difference was not significant either and we considered the values six hours after O_2_ inhalation as baseline again.

**Table 1:** Nociception specific blink reflex variables at baseline and during high flow oxygen inhalation (12 L/min)

Nociception specific blink reflex variable	Baseline	During high flow oxygen inhalation	
	Mean (SD)^a^	Mean (SD)^a^	*p*-value
R2 latency, symptomatic side (ms)			
Ipsilateral stimulation	44.95 (5.65)	47.09 (6.29)	0.225
Contralateral stimulation	48.75 (6.53)	50.56 (6.03)	0.392
Shortest R2 latency, symptomatic side (ms)			
Ipsilateral stimulation	39.05 (6.55)	43.37 (6.19)	0.025
Contralateral stimulation	44.12 (6.69)	44.65 (4.16)	0.789
R2 amplitude, symptomatic side (mV)			
Ipsilateral stimulation	0.27 (0.10)	0.25 (0.08)	0.387
Contralateral stimulation	0.19 (0.07)	0.17 (0.07)	0.235
R2 duration, symptomatic side (ms)			
Ipsilateral stimulation	52.63 (14.39)	50.36 (13.65)	0.263
Contralateral stimulation	47.74 (19.17)	45.74 (14.27)	0.381
R2 area, symptomatic side (mVms)			
Ipsilateral stimulation	2.28 (1.03)	2.04 (0.94)	0.202
Contralateral stimulation	1.57 (0.78)	1.25 (0.47)	0.132
R2 latency, asymptomatic side (ms)			
Ipsilateral stimulation	47.62 (10.60)	46.01 (8.96)	0.223
Contralateral stimulation	49.93 (9.44)	48.85 (7.51)	0.418
Shortest R2 latency, asymptomatic side (ms)			
Ipsilateral stimulation	42.92 (10.73)	40.18 (9.40)	0.086
Contralateral stimulation	44.17 (10.21)	42.58 (8.51)	0.484
R2 amplitude, asymptomatic side (mV)			
Ipsilateral stimulation	0.32 (0.15)	0.27 (0.11)	0.100
Contralateral stimulation	0.19 (0.09)	0.16 (0.09)	0.174
R2 duration, asymptomatic side (ms)			
Ipsilateral stimulation	49.29 (19.30)	53.80 (16.54)	0.216
Contralateral stimulation	47.97 (18.11)	49.50 (16.53)	0.394
R2 area, asymptomatic side (mVms)			
Ipsilateral stimulation	2.40 (1.13)	2.26 (0.86)	0.544
Contralateral stimulation	1.61 (0.82)	1.32 (0.64)	0.068

^a^All variables were normally distributed

### Discussion

In this study on the pathophysiology of CH using two-hourly transcutaneous stimulation sequences on the supraorbital nerves to elicit the nBR, none of the included patients did experience a CH attack during study participation, This may be an important serendipitous discovery for future prophylactic treatment studies, which we have discussed before [5].

Based on the nBR parameters there is no significant effect of O_2_, immediately and over time. There is also no significant difference between the symptomatic and asymptomatic side of the nBR parameters during the active phase of CH, but outside CH attacks. The stringent correction for multiple testing poses a risk for false negative results. Using no correction, however, none of the results (except for the ‘ipsilateral shortest R2 latency symptomatic side during O_2_ administration’ and ‘contralateral area asymptomatic side after six hours’) would have been significant.

It would be interesting to observe what will happen at brainstem level during CH attacks in humans. However, if noninvasive nociception specific supraorbital nerve stimulation (SNS) indeed is confirmed to act in a prophylactic way in CH, it may be difficult to measure nBR parameters during a CH attack.

The nBR was first studied in healthy subjects using a custom built concentric planar stimulating electrode allowing only the nociception specific A-delta fibers to be stimulated [3]. The nBR was further characterized in 104 healthy volunteers without any history of headache. Mean R2 onset latencies were 44.7 ms ipsilateral and 45.4 ms contralateral [6]. We are the first to present nBR reference values in CH patients and the effect of O_2_ on the nBR parameters. Consequently, it is not possible to make an accurate comparison with other nBR studies.

Our results of the nBR in CH and those from the literature raise some concerns about the applicability of the BR in CH. We searched the literature for BR R2 parameters and found conflicting results with studies indicating no difference between CH patients and healthy controls [7], a decreased excitability in CH patients based on a lower R2 amplitude [8], or an increased excitability based on an increased R2 duration and amplitude [9].

If we combine these variable findings with our own results of the nBR, we feel that the nBR may not be a suitable instrument for exploring the pathophysiology of CH, although a previous BR study suggested otherwise [10]. We have to emphasize that most studies measured conventional non-nociceptive BRs, nevertheless without consistent results. We studied a fairly small homogeneous group of ten male CH patients. It is desirable to study a larger population with both male and female patients comparing CH patients in the active vs the remission phase. Also, the addition of healthy controls is necessary to compare values between groups in further studies.

We conclude that the nBR is not different between symptomatic and asymptomatic sides in patients during the active phase of CH, outside of CH attacks, and that there is no measurable effect of O_2_ inhalation. Considering our observations with respect to the possible prophylactic action of SNS [5], it is questionable whether it will ever be possible to accurately measure the nBR during CH attacks.

## Abbreviations

BR: Blink reflex
CH: Cluster headache
L/min: Liter/minute
nBR: Nociception specific blink reflex
O_2_: Oxygen
pps: Pulse per second
R1: Early pontine response
R2: Late medullary response
SNS: Supraorbital nerve stimulation
